# Sugar cane extracts as natural taste modulators: potential for sugar reduction in beverages and beyond

**DOI:** 10.3389/fnut.2025.1603101

**Published:** 2025-05-20

**Authors:** Thalia Marina Llalla Vidal, Gregor Macnab, Shane Mitchell, Matthew Flavel

**Affiliations:** ^1^The Product Makers, Melbourne, VIC, Australia; ^2^Poly Gain Pte Ltd., Singapore, Singapore; ^3^La Trobe University, Melbourne, VIC, Australia

**Keywords:** sugar cane extract, taste modulation, sweetness enhancement, polyphenols, mouthfeel, clean label, sugar reduction, natural flavorings

## Abstract

The reduction of added sugars in food and beverage products has become a global health priority, driven by rising rates of obesity, diabetes, and cardiovascular disease. Yet, achieving sugar reduction without compromising taste remains a major challenge for manufacturers. This review explores the potential of sugar cane extracts—specifically Modulex™, as natural taste modulators capable of enhancing sweetness, masking bitterness, and improving mouthfeel in reduced-sugar formulations. Derived from *Saccharum officinarum*, these extracts contain a complex mixture of sugars, polyphenols, amino acids, and minerals that act through multimodal sensory pathways, including interactions with sweet (T1R2/T1R3) and bitter (TAS2R) taste receptors. Evidence from sensory studies demonstrates that sugar cane extracts can significantly improve the flavor profile and consumer acceptability of beverages sweetened with natural or artificial low-calorie sweeteners. This review discusses the biochemical basis of these effects, their regulatory positioning, and their implications for product development aligned with clean-label trends and public health goals. Sugar cane extracts represent a promising ingredient for next-generation sugar reduction strategies that balance health, taste, and consumer preference.

## Introduction

Obesity and metabolic diseases are rapidly escalating global health crises. While the etiology of these conditions is multifactorial and complex, numerous studies have identified excessive sugar consumption as a major contributing factor (1). The consumption of added sugars in beverages has been particularly implicated in the rising incidence of type 2 diabetes, obesity, cardiovascular disease, and other metabolic disorders (2). Beyond the public health implications, this growing crisis imposes a significant burden on healthcare systems worldwide (3). In response, the World Health Organization (WHO) issued guidelines in 2015 recommending that free sugars constitute no more than 10% of daily energy intake, with a conditional recommendation to further reduce intake to 5% (approximately 25 g per day) (4).

Governments are addressing this challenge through various measures, including the implementation of taxes on sugar-sweetened beverages, front-of-pack labeling, and voluntary reformulation targets aimed at reducing sugar content in food and beverages (5). While these policies seek to mitigate excessive sugar consumption, they also underscore the pressing need for better alternatives that can replicate the sensory experience of sweetness without the associated negative health impacts. Consequently, food and beverage manufacturers face increasing pressure to develop lower-sugar or sugar-free products that still meet consumer expectations (6).

Consumer surveys indicate that approximately 60% of consumers prefer lower-sugar versions of their favorite drinks, provided the taste remains comparable to full-sugar counterparts (2). While sweeteners such as stevia provide a natural, calorie-free alternative to sugar, they often introduce off-flavors or unbalanced taste profiles (7). Furthermore, sugar is not solely a sweetener; it plays a key role in the overall flavor balance, mouthfeel, color, and preservation of foods. Reducing or eliminating sugar can lead to undesirable taste shifts, such as excessive sourness or bitterness, and alterations in texture and body (8). This creates significant challenges for developing beverages that replicate the sensory attributes of their full-sugar equivalents.

In addition to this issue are consumer preferences, which suggest that while many individuals are seeking healthier, lower-sugar options, the taste remains the critical factor in repeat purchases. As a result, food and beverage companies are increasingly exploring taste modulation strategies to enhance the flavor profile of reduced-sugar products. One such approach involves the use of flavor-modifying properties (FMPs), ingredients that can enhance sweetness, mask bitterness, or improve mouthfeel, to create a more sugar-like taste experience (9). The synergistic use of these modulators with non-nutritive sweeteners can help simulate the sensory perception of sugar, as it is known that there are distinct sensory pathways that respond to caloric sugars, contributing to the overall roundness of flavor and minimizing off-notes (10). In this context, sugar cane extracts have emerged as a promising solution (11).

Sugar cane extracts are derived from *Saccharum officinarum*, retaining the intrinsic sweetness and flavor compounds of the cane while minimizing the caloric sugar content. These extracts are typically obtained from sugar cane juice or molasses, a by-product of sugar refining, through specialized processing techniques designed to concentrate non-sugar phytochemicals. Rich in polyphenols, minerals, and organic acids, sugar cane extracts offer a complex chemical composition that has the potential to modulate taste perception. In addition to small amounts of natural sugars such as sucrose, glucose, and fructose, they contain amino acids, salts, and polyphenolic compounds. This unique composition has led to increasing research into the use of sugar cane extracts as natural taste modulators capable of replicating some of the sensory attributes of sugar (12). This review will explore the characteristics of sugar cane extracts and their role in sweetness modulation, from their production and chemical composition to their applications in food and beverage products, their synergy with sweeteners, and their alignment with current market and regulatory trends.

## Description of sugar cane extracts

Sugar cane extracts encompass a variety of ingredients derived from the juice of the *Saccharum officinarum* plant, processed to capture not only the inherent flavor but also the bioactive compounds present in the cane, beyond pure sucrose. In contrast to refined sugar, which is approximately 99% sucrose (12) these extracts contain a broader array of phytochemicals naturally occurring in sugar cane.

Importantly, sugar cane extracts are not classified as high-intensity sweeteners. Their sweetness arises primarily from residual natural sugars and potentially from sweet-tasting glycosides, including conjugates such as glucosides, galactosides, galacturonides, ethers, esters, arabinosides, sulfates, phosphates, xylose, arabinose, and aldohexoses. Modulex™ contains intrinsic sucrose, glucose, and fructose derived from the sugar cane plant, which contribute to its sweetness profile, in addition to a variety of other bioactive components, such as minerals (e.g., potassium), organic acids, amino acids, peptides, proteins, vitamins, and additional minerals.

Specific compounds found in sugar cane extracts may include, but are not limited to, sucrose, glucose, galactose, xylose, ribose, mannose, rhamnose, fructose, maltose, lactose, maltotriose, xylopyranose, raffinose, 1-kestose, theanderose, 6-kestose, panose, neo-kestose, nystose, glucans, and xylans. Furthermore, the extract may also contain dietary fiber, either naturally present from the extraction process or added during formulation.

Moreover, Modulex™ contains a range of polyphenol concentrations, from approximately 15 g/L to 400 mg/g. These polyphenols include, but are not limited to, syringic acid, chlorogenic acid, caffeic acid, vanillin, sinapic acid, p-coumaric acid, ferulic acid, gallic acid, vanillic acid, diosmin, diosmetin, apigenin, vitexin, orientin, homoorientin, swertisin, tricin, catechin, catechin gallate, epicatechin, quercetin, kaempferol, myricetin, rutin, schaftoside, isoschaftoside, and luteolin. Table 1 highlights some of the main components responsible for its benefits and characteristics (13).

**Table 1:** Main components identified in Modulex™ (34).

Compound	Range
Amino acids	5,000–15,000 μg/g
Potassium	14,000–22,000 mg/kg
Sodium	900–14,00 mg/kg
Calcium	200–300 mg/kg
Magnesium	1,400–2,100 mg/kg
Iron	0.3–0.7 mg/kg
Zinc	1.1–1.7 mg/kg
Selenium	<0.05 mg/kg
Chromium	<0.05 mg/kg

## Mechanisms of taste modulation

### Sweetness

All compounds that generate a sweet sensation bind to the G-protein-coupled receptors T1R2 and T1R3. Sweet taste receptors are activated not only by sugars (glucose, fructose, sucrose, maltose) but also by sweet amino acids, sweet proteins, and artificial sweeteners. However, not all sweeteners bind to the same site on the receptor. The sweet receptor contains multiple binding sites, and the binding site for each sweetener is dependent on its specific biochemical conformation. This variability in binding leads to distinct activation patterns of the sweet receptor (14). Each T1R subunit comprises three principal domains: an extracellular venus-flytrap domain, a seven-transmembrane spanning domain at the C-terminus, and a cysteine-rich linker joining both domains. Sugar cane extracts can influence taste perception through several mechanisms (15). Their composition, which includes polyphenols, peptides, proteins, minerals, polysaccharides, and oligosaccharides, enables them to interact with various domains of the T1R2/T1R3 receptors (14). Additionally, while the canonical pathway for sweet perception involves T1R2/T1R3, there is a hypothesis that other independent pathways may exist, such as those involving T1R3 homodimers or distinct downstream signaling mechanisms associated with natural sugars, in contrast to artificial sweeteners, potentially offering a more complete sweet perception (10).

### Bitterness

Another important mechanism is the suppression of bitterness and the reduction of metallic notes in products. Sugars, through their interaction with the central gustatory pathway, can alter the sensory perception of bitterness, suppressing it and enhancing sweetness. For instance, sugars like sucrose can effectively reduce the sensation of bitterness by competing with bitter taste molecules, thereby overshadowing bitterness through the reduced activation of TAS2R receptors and enhanced activation of T1R2/T1R3 receptors (16). Studies have shown that sucrose has been used effectively to suppress bitterness in both children and adults when exposed to compounds like caffeine, quinine, and urea (17).

While previous investigations have primarily focused on the interactions between T1R2/T1R3 sweet receptors, recent findings indicate that bitterness may also interact with a family of receptors, including the previously unidentified TAS2R receptors. Notably, most sweeteners, except sucrose, fructose, and glucose, tend to exhibit a bitter taste. It is only in recent years that a fraction of the biochemical mechanisms underlying taste perception have been unveiled (16).

### Mouthfeel

Mouthfeel refers to the term which links different physical or chemesthetic sensations in the mouth during the consumption of food or beverages, including viscosity, astringency, smoothness, creaminess, and mouth coating (18, 19). These sensations are mediated by chemical and mechanical receptors such as the calcium-sensing receptor (CaSR), transient receptor potential channels, proton-sensitive ion channels and potassium channels, which contribute to complex oral perceptions like kokumi. Compounds that improve or positively modulate this sensations are known as mouthfeel enhancers (19).

Sugar cane extracts contribute to mouthfeel enhancement. The presence of oligosaccharides, soluble fiber, or glycerol in some extracts can increase the body or thickness of low-sugar beverages (13, 20). Although present in small quantities, these components, in conjunction with mineral content (potassium, sodium and calcium), provide a sugar-like mouthfeel that is often lacking in non-sugar beverages (21). The mineral content helps activate the calcium-sensing receptor (CaSR), which is associated with kokumi flavor.

**Figure 1:**
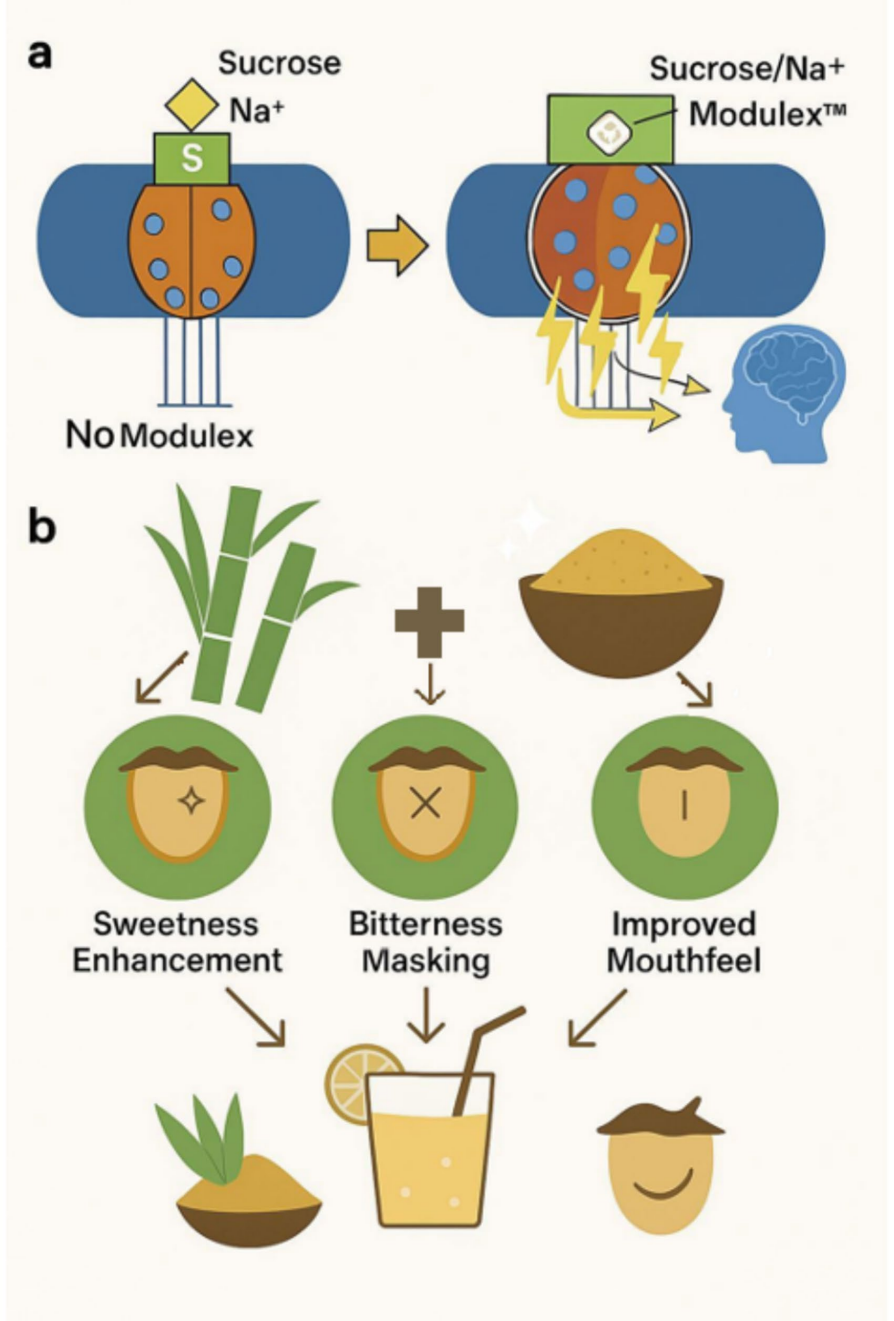
Proposed mechanism and functional sensory effects of Modulex™, a sugar cane derived flavor modulator. **(a)** Taste Receptor Mechanism: In the absence of Modulex™, binding of sucrose (yellow hexagon) to the sweet taste receptor result in a relatively weak activation and limited signal transduction, leading to a baseline sensory signal. In contrast, the presence of Modulex™ facilitates an allosteric enhancement of the receptor response alternative sweeteners. This is indicated by multiple bright yellow lightning bolts, symbolizing amplified sweet signal transduction and an enhanced perception of sweetness. **(b)** Sensory benefits of Modulex™: The benefits of using natural sugar cane extract Modulex™ as a taste modulator include sweetness enhancement, bitterness masking, through selective interaction with TAS2R, and improved mouthfeel. Together, these effects contribute to a more pleasant and balanced taste delivery in reduced sugar formulations that use alternative sweeteners.

In summary, sugar cane extracts modulate taste through a combination of sensory pathways: direct sweet receptor activation (from sugars), allosteric enhancement of sweetness (postulated for certain polyphenols and amino acids that may interact with sweet receptors or signaling pathways) (Figure 1a) (22), balanced sweetness, bitterness masking, and mouthfeel improvements (mineral content). These multimodal effects contribute to recreating a more sugar-like taste experience (Figure 1b).

### Applications in food and beverages

From non-alcoholic and alcoholic beverages to dairy products, sauces, confectionery, and baked goods, Modulex™ adapts seamlessly across multiple applications. It retains its effectiveness and stability across diverse formulations, making it a reliable choice for food and beverage manufacturers looking to innovate without sacrificing flavor (13).

Extensive testing of Modulex™, in combination with sweeteners like acesulfame potassium, aspartame, sucralose, and stevia, has shown promising results across a variety of beverages—including peach tea, fruit juices, electrolyte drinks, energy drinks, coffee-based beverages, and dairy drinks. These trials revealed significant reductions in metallic aftertaste and lingering sweetness while simultaneously enhancing the body and upfront sweetness of the drinks (13).

### Synergy with natural sweeteners

Modulex™ offers manufacturers the ability to reduce sugar content by up to 20–30%, significantly lowering calorie counts without compromising taste. When combined with low doses of sweeteners such as stevia, sucralose, acesulfame potassium, or aspartame, Modulex™ enhances their flavors while effectively masking any lingering bitterness or metallic aftertaste (13).

A recent sensory evaluation study compared the taste profiles of two popular beverage formulations—peach tea and orange carbonated soft drink (CSD)—with and without the addition of 0.1% Modulex™. The study assessed various sensory attributes, including likeability, metallic/astringent notes, upfront sweetness, lingering sweetness, and body (13).

**Figure 2:**
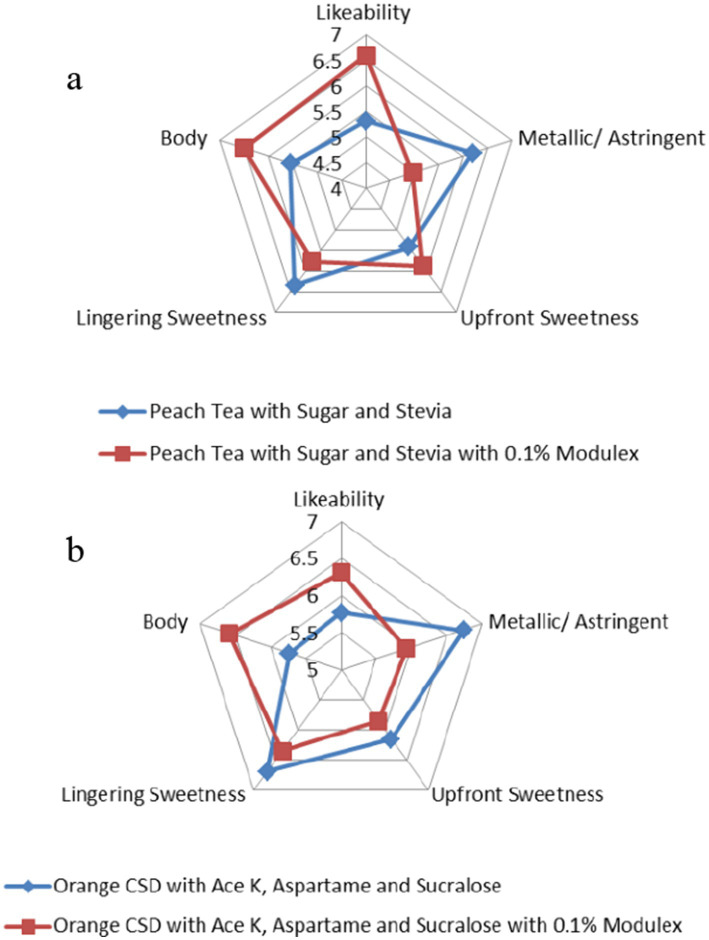
Spidergrams of sensory evaluation for two beverage types with and without the addition of sugarcane extract (Modulex™): **(a)** peach tea sweetened with sugar and stevia, and **(b)** orange carbonated soft drink (CSD) sweetened with acesulfame potassium (Ace K), aspartame, and sucralose. Sensory attributes assessed included likeability, metallic/astringent notes, upfront sweetness, lingering sweetness, and body. The addition of 0.1% Modulex™ (red line) improved overall likeability, enhanced body, and reduced metallic/astringent perception compared to the control formulations without Modulex™ (blue line).

In the first evaluation, researchers tested a standard peach tea formulation sweetened with sugar and stevia against a modified version containing 0.1% Modulex™. The results revealed a clear improvement in likeability, with the test sample scoring 6.58, compared to 5.31 for the standard version (13). The metallic/astringent quality, often associated with stevia (23), was significantly reduced by 20% in the test formulation. Additionally, the test version showed a slight increase in upfront sweetness (9%) and body (17%), while lingering sweetness was reduced by 10% (Figure 2a) (13).

A second evaluation focused on an orange CSD sweetened with acesulfame potassium, aspartame, and sucralose. Like the peach tea study, a test formulation containing 0.1% Modulex™ was compared against the standard. The test sample again demonstrated a higher likeability score by 10% and increased body perception by 15% (13). The metallic/astringent perception, commonly linked to artificial sweeteners (24, 25), decreased by 14%. Moreover, both upfront and lingering sweetness were reduced by 8 and 5%, respectively (Figure 2b) (13).

### Analysis

#### Consumer attitudes toward sugar cane extracts

For any new food ingredient to succeed, consumer acceptance is crucial. Sugar cane extracts benefit from a generally positive perception due to their natural origin. Consumers tend to view ingredients derived from fruits or plants more favorably than synthetic additives (26). In this regard, sugar cane extract can be seen as an extension of a familiar source (sugar cane), which may alleviate concerns compared to unfamiliar sweetener chemicals (27). The ability to label these extracts as “natural flavor” or “sugar cane extract” aligns them with the clean label movement, which is gaining traction among consumers. Recent surveys indicate that 67% of consumers seek clean labels; the presence of “sugar cane” on the label may evoke a sense of wholesomeness or at least neutrality (28, 29).

Another aspect influencing consumer attitude is taste experience (30). When used effectively, sugar cane extracts have the potential to improve the taste of low-sugar products, which could lead to better consumer acceptance. Studies suggest that incorporating Modulex™ into artificially or naturally sweetened beverages can enhance overall product likability by reducing metallic and astringent notes while improving body. This improved balance in taste perception makes the beverages more enjoyable, leading to a consistent preference for products containing Modulex™. Consequently, consumer attitudes toward lower-sugar products containing sugar cane extracts are likely to be positive, especially when these products are perceived as more enjoyable and flavorful (13).

One potential challenge could be a misunderstanding of the term “sugar cane extract.” Some consumers may mistakenly believe it is merely sugar. Educating consumers about the beneficial antioxidants and bioactive compounds in sugar cane extracts, as opposed to just sugar, could further enhance acceptance (31).

## Discussion

Sugar cane extracts are emerging as natural taste modulators that address a key challenge in the food and beverage industry: reducing sugar content without compromising sensory quality. The evidence presented in this mini-review highlights that these extracts can play multiple roles, from adding a hint of sweetness to masking bitterness and enhancing mouthfeel, thereby effectively recreating the sensory experience of sugar in reduced-sugar formulations. Unlike single-compound intense sweeteners, sugar cane extracts offer a multifaceted approach to taste modulation due to their complex composition of sugars, acids, and polyphenols. This complexity provides a significant advantage in formulating palatable low-sugar products, as it contributes to a more balanced and rich flavor profile. In practical terms, sugar cane extracts function as flavor enhancers or enablers that work synergistically with both nutritive and non-nutritive sweeteners to deliver a satisfying sweetness. They exemplify how leveraging natural plant constituents can help overcome some of the flavor challenges encountered when sugar is removed from formulations (13).

The significance of sugar cane extracts is further underscored by the evolving policy and health landscape. With organizations such as the WHO and various national governments—including those of Mexico, the United Kingdom, South Africa, Portugal, Chile, the Philippines, and Hungary (32)—setting aggressive sugar reduction targets, manufacturers are under increasing pressure to reformulate products (33). Ingredients like sugar cane extracts provide a valuable tool for achieving these public health objectives by enabling sugar reduction without a drastic decline in product acceptability. In essence, sugar cane extracts help align the food industry’s reformulation efforts with health policies: products can meet nutritional criteria (lower sugar, lower calories) while still meeting consumer preferences for great taste. This alignment is crucial for translating policy measures into meaningful dietary changes. For instance, if sugar taxes make full-sugar beverages more expensive, consumers will only shift to alternatives if those alternatives taste good. Sugar cane extracts can improve the flavor profile of these alternatives, thereby indirectly supporting the effectiveness of sugar-reduction policies (13).

In conclusion, sugar cane extracts represent a promising strategy in the ongoing effort to reduce added sugars in the food supply while maintaining palatability. They harness the natural sweetness and complexity of the sugar cane plant to modulate taste in ways that consumers find acceptable, and in some cases, indistinguishable from full-sugar products. Their dual role in contributing functional health benefits, such as antioxidant properties and lower glycemic response, further enhances their potential for broader application. As the food industry responds to public health imperatives and consumer demand for healthier products, sugar cane–derived taste modulators are positioned to become an important part of formulators’ toolkits. By marrying natural ingredients with innovation, they exemplify how nutritional profiles can be improved without requiring consumers to sacrifice the enjoyment of their favorite foods and beverages. The continued uptake of sugar cane extracts in product reformulation efforts will serve as a telling indicator of their impact, potentially making reduced-sugar products the new norm rather than the exception in the marketplace.
